# A novel receptor – ligand pathway for entry of *Francisella tularensis *in monocyte-like THP-1 cells: interaction between surface nucleolin and bacterial elongation factor Tu

**DOI:** 10.1186/1471-2180-8-145

**Published:** 2008-09-12

**Authors:** Monique Barel, Ara G Hovanessian, Karin Meibom, Jean-Paul Briand, Marion Dupuis, Alain Charbit

**Affiliations:** 1 INSERM U570, Unité de Pathogénie des Infections Systémiques, Université Paris Descartes, Faculté de Médecine Necker Enfants-Malades, 156 rue de Vaugirard, 75730, Paris Cedex 15, France; 2 UPR 2228 CNRS, Régulation de la transcription et maladies génétiques, UFR Biomédicale des Saints-Pères, 45 rue des Saints Pères, 75270, Paris Cedex 06, France; 3 UPR 9021 CNRS, Immunologie et Chimie Thérapeutiques, Institut de Biologie Moléculaire et Cellulaire, 15 rue René Descartes, 67084, Strasbourg Cedex, France

## Abstract

**Background:**

*Francisella tularensis*, the causative agent of tularemia, is one of the most infectious human bacterial pathogens. It is phagocytosed by immune cells, such as monocytes and macrophages. The precise mechanisms that initiate bacterial uptake have not yet been elucidated. Participation of C3, CR3, class A scavenger receptors and mannose receptor in bacterial uptake have been already reported. However, contribution of an additional, as-yet-unidentified receptor for *F. tularensis *internalization has been suggested.

**Results:**

We show here that cell-surface expressed nucleolin is a receptor for *Francisella tularensis *Live Vaccine Strain (LVS) and promotes LVS binding and infection of human monocyte-like THP-1 cells. The HB-19 pseudopeptide that binds specifically carboxy-terminal RGG domain of nucleolin inhibits LVS binding and infection of monocyte-like THP-1 cells. In a pull-down assay, elongation factor Tu (EF-Tu), a GTP-binding protein involved in protein translation, usually found in cytoplasm, was recovered among LVS bacterial membrane proteins bound on RGG domain of nucleolin. A specific polyclonal murine antibody was raised against recombinant LVS EF-Tu. By fluorescence and electron microscopy experiments, we found that a fraction of EF-Tu could be detected at the bacterial surface. Anti-EF-Tu antibodies reduced LVS binding to monocyte-like THP-1 cells and impaired infection, even in absence of complement and complement receptors. Interaction between EF-Tu and nucleolin was illustrated by two different pull-down assays using recombinant EF-Tu proteins and either RGG domain of nucleolin or cell solubilized nucleolin.

**Discussion:**

Altogether, our results demonstrate that the interaction between surface nucleolin and its bacterial ligand EF-Tu plays an important role in *Francisella tularensis *adhesion and entry process and may therefore facilitate invasion of host tissues. Since phagosomal escape and intra-cytosolic multiplication of LVS in infected monocytes are very similar to those of human pathogenic *F. tularensis *ssp *tularensis*, the mechanism of entry into monocyte-like THP-1 cells, involving interaction between EF-Tu and nucleolin, might be similar in the two subspecies. Thus, the use of either nucleolin-specific pseudopeptide HB-19 or recombinant EF-Tu could provide attractive therapeutic approaches for modulating *F. tularensis *infection.

## Background

*Francisella tularensis *is a small non-motile Gram-negative bacterium that causes the zoonotic disease tularemia in large number of animals, such as rabbits, hares, and small rodents [1]. *F. tularensis *is also one of the most infectious human bacterial pathogens as ten bacteria can cause disease in humans [1,2]. Humans acquire infection by direct contact with sick animals, inhalation, ingestion of contaminated water or food, or by bites from ticks, mosquitoes or flies. *F. tularensis *has significant potential as an agent of bio-terrorism due to its infectivity and capacity to infect in form of aerosols and its ability to cause illness and death [1]. The two primary human pathogens are *F. tularensis *subspecies *tularensis *(type A strain) and *F. tularensis *subspecies *holarctica *(type B strain). *F. tularensis *live vaccine strain (LVS) is an attenuated type B strain [3]. Other subspecies (ssp) of *F. tularensis *exist: *F. tularensis *ssp *mediasiatica*, and *F. tularensis *ssp *novicida*. The four subspecies differ in terms of their pathogenicity and geographic origin, but are phylogenetically very closely related. *F. tularensis *is a highly virulent facultative intracellular bacterium, replicating intracellularly and disseminating within host mononuclear phagocytes. After entry into macrophages, *F. tularensis *initially resides in the phagosome, whose maturation is then arrested. After 2 hours of infection, the phagosome membrane is disrupted and the bacterium replicates freely in the cytoplasm of the macrophages [3,4].

While several molecular aspects of the intracellular life of *F. tularensis *after entry have been elucidated, the exact mechanisms that mediate uptake of this highly infectious bacterium are still not fully understood. Participation of C3 [5], CR3 [6], class A scavenger receptors [7] and mannose receptor [8] in bacterial uptake have been already reported. However, contribution of an additional, as-yet-unidentified receptor for *F. tularensis *internalization has been suggested [8].

In the present work, we evaluated whether nucleolin could serve as a cell surface receptor for LVS and mediate its binding and subsequent internalization. Indeed, nucleolin has been shown to be localized not only in the nucleus [9], but also on the cell surface and to mediate internalization of specific ligands, including HIV particles [10,11]. In response to binding of a ligand to surface nucleolin, ligand-nucleolin complexes become internalized, by an active process, therefore allowing intracellular import of the ligand. Nucleolin, expressed at the cell surface in a high molecular weight protein complex, is in close association with the intracellular actin cytoskeleton [12]. Participation of actin microfilaments in uptake of *F. tularensis *was supported by its inhibition by cytochalasin B [5,13]. Nucleolin is also involved in cell proliferation [9] and in activation of CD21 on B cells [14]. Of particular interest, nucleolin has been recently described as a receptor for the adhesin of *E. coli *O157:H7 [15,16].

We herein demonstrate that surface nucleolin present on human monocyte-like THP-1 cells is a functional receptor for LVS. Moreover, we demonstrate that the LVS bacterial ligand for human nucleolin is elongation factor Tu (EF-Tu). We also provide evidence that EF-Tu, a GTP-binding protein usually found in cytosol [17], is present in the LVS membranes and exposed on bacterial surface. Finally, we show that nucleolin expressed on the surface of human monocyte-like THP-1 cells interacts, through its C-terminal RGG domain, with the bacterial surface-exposed EF-Tu protein. This nucleolin – EF-Tu interaction plays an important role in *F. tularensis *LVS adhesion and entry process and may therefore facilitate invasion of host tissue.

## Methods

### Human cells, bacteria, antibodies and human serum

The human monocyte-like cell line THP-1 (ATCC^® ^Number: TIB-202™) was grown in suspension in RPMI containing fetal calf serum (FCS) at 37°C in a CO_2 _incubator. Cell viability was measured by Trypan Blue exclusion test. *F. tularensis *Live Vaccine Strain, LVS, was kindly supplied by A. Sjöstedt [18] and grown either in Schaedler broth containing vitamin K3 or on chocolate agar plates containing polyvitex (Biomerieux) at 37°C. LVS-GFP was obtained by transformation of LVS bacteria with pFNLTP6 *gro-gfp*, provided by Dr. Zahrt [19]. Antibodies (Abs) used were: polyclonal mouse anti-EF-Tu prepared in our laboratory, as described below; rabbit anti-IglA, kindly provided by Dr. Nano [20], anti-LVS (Becton Dickinson), anti-nucleolin (Abcam), anti-GST (Oncogene); monoclonal antibodies (MAbs) were anti-nucleolin (clone D3) [21], anti-glyceraldehyde phosphate dehydrogenase (GAPDH) (Santa Cruz Biotechnology) as an isotype-matched negative control for cell surface nucleolin and anti-CD11/CD18 (anti-CR3 receptors) (Abcam), which specifically recognizes the human CD11/18 activation epitope. Secondary antibodies were goat anti-rabbit (GAR) or goat anti-mouse (GAM) conjugated to HRP (Dako) for immunoblotting experiments or with Alexa Fluor cytochromes for microscopy experiments. Human serum AB (PAA) was handled in a manner to preserve complement activity [22]. Heat-inactivated (H.I.) serum was obtained by incubating serum for 30 min at 56°C.

### Peptide constructs

The synthesis of the 5 [Lys (CH_2_N)Pro-Arg]-template-assembled synthetic peptide referred to as HB-19 and the nine-D-arginine peptide construct, referred to as 9Arg, synthesized using Fmoc-protected D-Arg residue was as described previously [11,23]. The p63 peptide was synthesized according to the last 63 amino acid residues of human nucleolin (KGEGGFGGRGGGRGGFGGRGGGRGGRGGFGGRGRGGFGGRGGFRGGRGGGGDHKPQGKKTKFE) [24]. The synthetic F3 peptide is a tumor-homing peptide corresponding to the 34 amino acid fragment (AKVKDEPQRRSARLSAKPAPPKPEPKPKKAPAKK) of a high mobility group protein, HMG2N [25]. For the biotin-labeled p63 peptide, the biotin moiety was introduced during peptide assembly as an Fmoc Lys-biotin derivative at the N-terminus of the peptide. All peptides were obtained at a high purity (95%), and their integrity was controlled by matrix-associated laser desorption ionization-time-of-flight analysis [11].

### Preparation of subcellular fractions of LVS

After 24 h of culture in broth, LVS bacteria were collected by centrifugation and either directly lysed in BPER buffer (Novagen) or suspended in 50 mM Tris/HCl pH 8.0. Suspended bacteria were then disintegrated in a Fast-Prep FP120 Cell Disrupter (ThermoSavant) with Fast-Prep beads using 4 repeated cycles of 30 sec at a 6.5 speed. After 10 min on ice, undisrupted microbes were eliminated by centrifugation for 5 min at 2,800 × g. Supernatant was then centrifuged for 60 min at 105,000 × g in a Beckmann Optima Max ultracentrifuge, at 4°C. The pellet, which contained the bacterial membranes, was washed twice in TNE (50 mM Tris, 150 mM NaCl, 1 mM EDTA, pH 8.0) by centrifugation and then solubilized for 45 min at 4°C in TNE containing 1% NP40 and 1% Triton X100. A further centrifugation at 12,000 × g for 20 min at 4°C separated the soluble membrane proteins present in the supernatant from membrane-associated proteins present in the pellet. Protein concentration of different fractions was determined by the BCA assay (Pierce).

### Preparation of EF-Tu recombinant proteins and generation of murine polyclonal antibodies

LVS chromosomal DNA was purified with Turbogen kit (Invitrogen). PCR reactions were performed in Bio-Rad I Thermal Cycler with 100 pmoles/μl of 5' primer 5'-CCG*GAATTC*ATGGCTAAAGAAAAATTTGAGCGTTC-3' (nt 3141 to 3156) and 3' primer 5'CGA*GTCGAC*TTACTCGATAATTTTAGCTACAACACCAGCACC-3' (nt 3246 to 3261). The EF-Tu amplicon of 1.1 kbp was purified on agarose gel, cloned in pCR2-TOPO1 vector (Invitrogen) and sequenced in Institut Cochin (Genomic Sequencing Department). Once its sequence confirmed, EF-Tu was subcloned in pGEX-4T1 (Amersham Biosciences) or pET28a+ (Novagen) into *Eco*RI-*Sal*I sites, in DH5α. After transforming in *E. coli *strain BL21or BL21DE3, respectively, GST-fusion proteins and His-fusion proteins were produced as previously described [26] and purified either on glutathione-Sepharose 4B beads (Amersham Biosciences) or on Ni-column (Novagen), as described by manufacturers. His-fusion EF-Tu protein was used as immunogen for immunizing Balb/c mice. Non-immune serum (NIS) was obtained from mice injected only with Freund adjuvant.

### Pull-down assays

Pull-down assays were performed either with GST recombinant proteins or with biotin-avidin beads. For GST recombinant proteins, 5 μg of GST or GST-EF-Tu bound on glutathione-Sepharose beads were incubated with 500 μg human membrane proteins, obtained as previously described [14] by solubilizing THP-1 cell membranes with 1% NP40 in TNE, containing protease inhibitors (Roche Diagnostics). Biotin-avidin beads were used to characterize bacteria proteins bound on RGG p63 carboxy-terminal domain of nucleolin. For that purpose, 500 μg of LVS membrane proteins were incubated for 2 h at 8°C with different concentrations of the biotinylated p63 peptide. The complex formed between bacteria membrane proteins and biotinylated p63 peptide was isolated by purification using 100 μl avidin-agarose (ImmunoPure Immobilized Avidin, Pierce), after incubation for 2 h at 8°C. Specificity of the binding with the p63 domain was determined by preincubation of 500 μg of bacteria membrane proteins with unlabeled p63 peptide for 2 h at 8°C, before further incubation with the biotinylated p63 peptide for 2 h at 8°C. After extensive washes of the different samples, purified proteins were denatured by heating at 95°C for 10 min in sample buffer containing SDS and analyzed by SDS-PAGE and immunoblotting assays.

### Immunoblotting assays

Recombinant proteins, proteins from human cells or present in the different subcellular fractions of LVS, and proteins recovered from pull-down assays were submitted to SDS-PAGE. Separated proteins were electrotransferred on nitrocellulose membranes (Schleicher & Schuell), using a semi-dry Transblot apparatus (Bio-Rad). Membranes were incubated in Phosphate-buffered saline (PBS) containing 5% powdered milk and then with the indicated Abs. After washing in PBS/0.05% Tween-20 (PBS-T), HRP-linked secondary Abs were added. Nitrocellulose sheets were washed and binding of second Abs was detected using the ECL Plus kit (Amersham).

### Infection of human cells

Thirty minutes before infection, LVS bacteria were suspended at 37°C in RPMI containing 10% human serum AB, except when otherwise indicated. Human monocyte-like THP-1 cells were washed in RPMI, suspended in RPMI containing 5% FCS (RPMI-FCS) and infected with LVS for 30 min at 37°C, without centrifugation to promote bacteria-cell interaction [27] at a Multiplicity of Infection (MOI) of 100:1 (bacteria/cell). Cells were then washed with RPMI containing 10 μg/ml gentamicin to remove extracellular bacteria, then suspended at 0.7 × 10^6 ^per ml in RPMI-FCS with gentamicin. This point was referred to as time zero (T = 0). Cells were further incubated statically for indicated times at 37°C. At indicated times, 100 μl THP-1 cells were lysed for 15 min in distilled water. Then, serial dilutions were performed in 0.15 M NaCl and the number of intracellular bacteria present in cells was determined by plating 100 μl on chocolate agar plates. When indicated, THP-1 cells were pretreated before infection for 2 h at 37°C with anti-CR3 Mab, the different peptides described above or with cytochalasin D (Sigma), an actin polymerization inhibitor. In some experiments, THP-1 cells were also pre-incubated with recombinant His-EF-Tu protein. This recombinant protein was tested for its toxicity on human cells, which was found to be negative (data not shown). Adherent THP-1 cells or blood MDM plated at 2 × 10^5 ^per ml were infected as described above for THP-1 cells in suspension, except that number of intracellular bacteria was quantified after adding 1 ml H_2_O to each well before plating serial dilutions.

### Fluorescence and Confocal experiments

Binding of bacteria on human cells was analyzed by either fluorescence or confocal microscopy, at the Cell Imaging Facility (Faculté de Médecine Necker Enfants-Malades). For fluorescence microscopy, THP-1 cells were incubated either with RPMI, the different peptides or recombinant His-EF-Tu for 2 h at 37°C. They were then infected for 30 min at 37°C with LVS-GFP (MOI of 100:1), which had been pre-incubated either with RPMI, NIS or anti-EF-Tu for 30 min at 37°C. Cells were then washed in RPMI without gentamicin and fixed with 4% para-formaldehyde (PFA), as described below. Cells were visualized with a fluorescence microscope (Zeiss Axioplan 2) using Qimaging Digital Camera with Q Capture Pro Fluorescence analysis software. For experiments by confocal microscopy, THP-1 cells infected or not with LVS, for 30 min at 37°C, were washed in PBS/Ca^2+^Mg^2+ ^(Gibco) and suspended at 3 × 10^6 ^cells/ml. Cells were then incubated for 30 min at room temperature (RT) with 5% goat serum in PBS, to block non-specific binding sites, then for 45 min at RT with first Abs, at indicated dilutions. After washing, cells were incubated for 45 min at RT with second Abs diluted 1/100 (Alexa Fluor 488-labeled GAM and/or Alexa Fluor 546-labeled GAR) in the dark. After washing, cells were fixed with PFA for 15 min at RT and incubated for 10 min at RT with 50 mM NH_4_Cl to quench residual aldehydes. After centrifugation, the cell pellet was suspended in Mowiol and mounted on glass coverslips. Cells were visualized at the appropriate wavelengths under a Zeiss LSM 5 Pascal confocal microscope with argon (458/488 nm) and helium neon (543 nm) lasers with LSM analysis software. We present the photographs of cells with typical morphology and staining. For quantification by confocal microscopy, THP-1 cells were infected as described above except they were infected with LVS-GFP and then incubated with anti-nucleolin MAb, diluted 1/1000, followed by Alexa Fluor 568-labeled GAM. After fixation of cells with PFA for 15 min at RT and incubation with 50 mM NH_4_Cl, DAPI was added for 5 min in PBS containing 5% gelatin, before washing with PBS. Cell pellet, mounted on glass coverslip in Mowiol, was visualized with a Leica TCS SP5 confocal microscope with diode (405 nm), argon (458/488 nm), DPSS (561 nm) and helium neon (633 nm) lasers and LASAF program. Co-localization of LVS-GFP with nucleolin was measured on more than 500 cells using the "Co-localization" module of Imaris 5.0.2. software, which analyzes stacks of confocal sections, as previously described [28].

For direct labeling of LVS bacteria with different antibodies, a pellet of 2 × 10^9 ^LVS was processed as described above for labeling of human cells.

### Immunoelectron microscopy (IEM)

LVS bacteria grown on plates were harvested, suspended in PBS and centrifuged at 12,000 × g for 30 sec to form loose pellets. Supernatant was removed and 2% PFA-0.1% glutaraldehyde in 0.1 M Millonig's phosphate buffer (MPB) was added to each pellet for 2 h. After rinsing three times with MPB, the pellets were then processed for negative-contrast IEM (nc-IEM) as previously described [29], except binding of the first antibodies diluted 1/200 was detected with a 1/30 dilution of protein A labeled with 10-nm gold spheres (Aurion, The Netherlands). The specimens were examined at 80 kV with a JEM-100CXII electron microscope (JEOL).

### FACS analysis

THP-1 cells uninfected or infected by LVS, as described above, were incubated with different antibodies for 45 min at RT. After washing, THP-1 cells were incubated with Alexa Fluor 488-labeled second antibodies in dark place, for 45 min at RT. After washing, THP-1 cells were fixed with PFA and incubated in NH_4_Cl. Cells were then centrifuged and processed for analysis by FACS scan flow cytometer (Becton Dickinson). For each sample, 10,000 viable cells were gated following size (forward scatter, FSC) and granularity side scatter (SSC) and analyzed with Cell Quest™ Software (Becton Dickinson).

### Preparation of C3bi-coated erythrocytes (EC3bi) and their binding on THP-1 cells

Sheep erythocytes coated with IgM (EA) were kindly provided, by Dr. V. Fremeaux-Bacchi (Hopital Européen Georges Pompidou, Paris). EC3bi were prepared by incubating EA in 10% C5-deficient serum (Sigma) for 60 min at 37°C, as previously described [30]. EC3bi were incubated with THP-1 cells for 60 min at RT and percentage of cells forming rosettes was determined by counting 100–200 cells in a microscope hemacytometer chamber. Prior to incubation with EC3bi, THP-1 cells were incubated either with RPMI, 10 μM HB-19 or 9R peptides or 20 μg anti-CR3 Mab for 2 h at 37°C. A rosette was defined as a cell with two or more adherent sheep erythrocytes.

### Statistical analyses

Binding data were expressed as the mean ± SD. Student's t test was used to determine the statistical differences between the various experimental and control groups. A p value of < 0.01 was considered to be statistically significant.

## Results

### Nucleolin expressed on monocyte-like THP-1 cells cell surface is down-regulated by LVS infection

**Figure 1:**
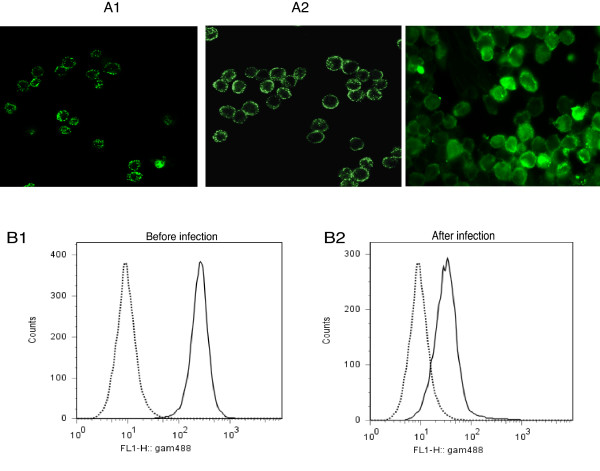
**Down-regulation of cell surface nucleolin on THP-1 monocyte-like cell surface after LVS infection**. **A**: THP-1 cells in suspension were incubated first with anti-nucleolin either polyclonal (**part A1**) or MAb (**part A2**), diluted 1/200 and then treated with PFA. In **part A3**, THP-1 cells were first treated by PFA and then incubated with polyclonal anti-nucleolin Ab. Analysis was performed by fluorescence microscopy at 63× magnification. **B: **FACS analysis of nucleolin expression on cell surface of uninfected cells (**B1**) or cells infected for 30 min by LVS (**B2**). THP-1 cells were incubated either with anti-GAPDH as isotype negative control (**dotted line**) or with anti-nucleolin (**solid line**) MAbs. These experiments are representative of five different experiments.

We first verified the presence of nucleolin at the surface of THP-1 cells by fluorescence microscopy analysis using polyclonal and monoclonal antibodies specific for human nucleolin [12,21] (Fig 1A). When cells were first incubated with specific and secondary antibodies before treatment with PFA, both polyclonal (Fig 1A1) and monoclonal (Fig 1A2) anti-nucleolin Abs labeled THP-1 cell surface, as previously described [12]. When cells were first treated by PFA and then incubated with the different antibodies, only staining of cytoplasm was observed, most likely due to permeabilization of the cell membrane and entry of the antibody into the cytosol (Fig 1A3). Therefore, all subsequent fluorescent experiments were performed, by incubating cells with antibodies before treatment with PFA. We also monitored the changes in cell surface nucleolin expression upon LVS infection by FACS analysis (Fig 1B). Fluorescence intensity of cell surface expressed nucleolin decreased from 5 × 10^2 ^± 1 × 10^2 ^A.U. (arbitrary units) in uninfected cells (Fig 1B1) to 5 × 10^1 ^± 0.5 × 10^1 ^A.U., when cells were infected by LVS (Fig 1B2).

### Nucleolin mediates binding of LVS and infection of human monocyte-like THP-1 cells

We evaluated the role of nucleolin in LVS binding and infection of human monocyte-like THP-1 cells, by testing the effect of the pseudopeptide HB-19. This pseudopeptide specifically and irreversibly binds cell-surface expressed nucleolin through its RGG domain located on the C-terminal tail [11,24]. We used THP-1 cells in suspension, *i.e*. in their monocyte-like state, in presence of human serum AB, as opsonization of LVS by human complement has been shown to enhance its ability to infect human cells [5].

**Figure 2:**
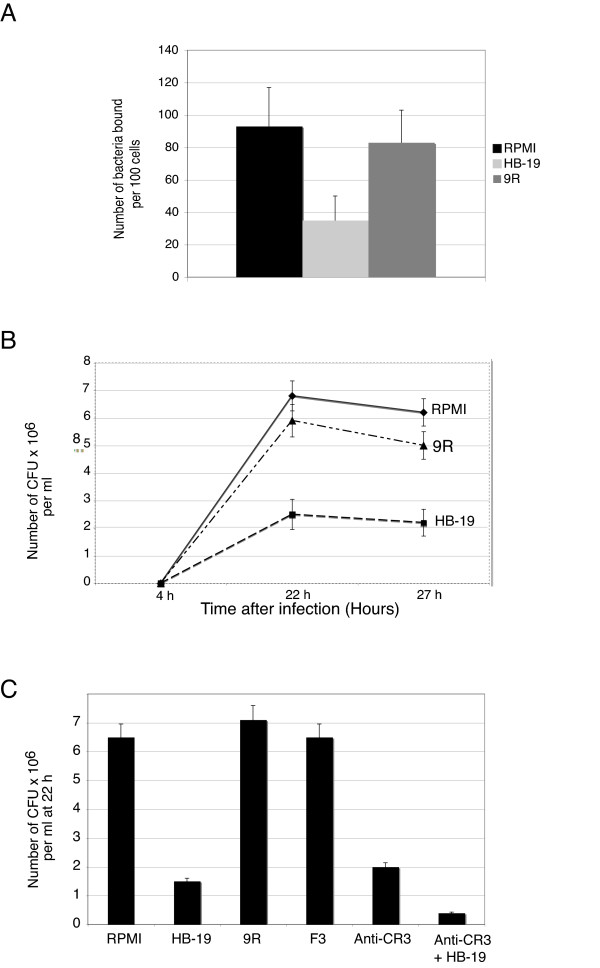
**Interaction of nucleolin with LVS is involved in binding and infection of human monocyte-like THP-1 cells**. **A**: THP-1 cells were incubated in RPMI, in absence or in presence of 5 μM HB-19 pseudopeptide or 9R control peptide. Opsonized LVS transformed with GFP expressing plasmid (LVS-GFP) were then added for 30 min. THP-1 cells were analyzed by fluorescence microscopy. Fifteen fields containing average of 100 cells were examined to quantify number of bacteria bound on human cells. Data show mean from three independent experiments ± SD values indicated as error bars. * = p < 0.01, indicates significant difference for HB-19-treated group compared to control group with 9R peptide. **B**: Intracellular replication of LVS. THP-1 cells were incubated with RPMI, 5 μM HB-19 or 9R before addition of opsonized LVS for 30 min. Cells were washed with RPMI containing gentamicin to kill extracellular bacteria and incubated in RPMI-FCS and gentamicin for indicated times. Quantification of intracellular bacteria was performed as described in Methods. Results show mean from three independent experiments, each performed in triplicate ± SD values indicated as error bars. **C**: THP-1 cells were incubated either with RPMI, 10 μM HB-19 alone or in presence of anti-CR3 Mab, 10 μM 9R or F3, or anti-CR3 Mab. Opsonized LVS were added for 30 min. Cells were washed with RPMI containing gentamicin and incubated in RPMI-FCS and gentamicin for 22 h. Quantification of intracellular bacteria was performed as described in Methods. Results show mean from five independent experiments, each performed in triplicate ± SD values indicated as error bars.

Effect of HB-19 on LVS binding on THP-1 cells was followed using fluorescent microscopy. We counted the number of LVS bacteria expressing GFP (LVS-GFP) bound per 100 human THP-1 cells in fifteen different fields (Fig 2A). The mean number of bacteria bound per cell was almost one bacterium per cell, a value consistent with previous reports on monocytes or macrophage-derived monocytes (MDM) [5,6]. Pre-incubation of THP-1 cells with HB-19 resulted in a 63% decrease in the number of bound LVS-GFP bacteria. We used as a negative control, the protease-resistant basic 9Arg peptide (9R) that does not interact with nucleolin. As expected, this peptide did not significantly affect the number of bacteria bound on THP-1 cells.

We then analyzed effect of HB-19 treatment on infection by LVS. At 4 h, the 4 ± 0.5 × 10^4 ^CFU/ml obtained in presence of RPMI were decreased to 1.5 ± 0.2 × 10^4 ^CFU/ml in presence of 5 μM HB-19. Maximum of intracellular bacterial multiplication (7 × 10^6 ^CFU/ml) was reached at 22 h (Fig 2B), corresponding to average of 11 intracellular bacteria per cell. When THP-1 cells were pre-incubated with 5 μM HB-19, a 63% decrease in LVS infection was observed at 22 h. The strongest inhibitory effect of infection at 22 h was observed with a concentration of 10 μM HB-19 (evaluated over a range of concentration from 1 to 10 μM HB-19, data not shown). Therefore, all following experiments were performed with 10 μM HB-19, with number of intracellular bacteria at 22 h decreased by 78%, (*i.e. *yielding to average number of 2 bacteria per cell) (Fig 2C). 9R peptide had no significant effect on LVS infection of THP-1 cells. As HB-19 pseudopeptide forms an irreversible complex with surface nucleolin by binding to RGG carboxy-terminal domain [24], we also evaluated effect of F3 peptide that binds acidic domains localized at N-terminal part of nucleolin [25]. F3 had no effect on bacterial infection (Fig. 2C).

Involvement of CR3 receptors in uptake of LVS though complement component has been largely described [6,31] and prompted us to monitor the possible combined effect of HB-19 and anti-CR3 MAb on the level of bacterial infection of THP-1 cells (Fig 2C). Anti-CR3 alone induced 70% decrease in infection, in agreement with previous observations [7]. Strikingly, incubation of THP-1 cells with both HB-19 and anti-CR3 MAb led to 95% decrease in the level of infection (lowering the number of intracellular bacteria in THP-1 cells from 3 to less than 0.6 bacteria per cell).

**Figure 3:**
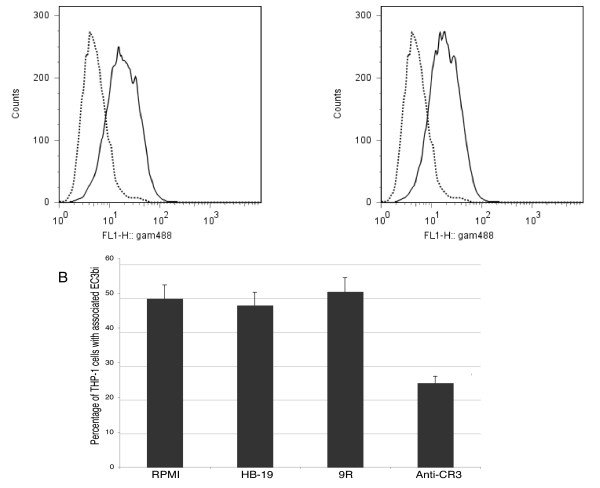
**Expression of CR3 is not affected by HB-19**. **A**. FACS analysis of CR3 expression on surface of THP-1 cells preincubated with 10 μM HB-19 (**A1**) or 10 μM 9R (**A2**). THP-1 cells were incubated either with anti-GAPDH as negative control (**dotted line**) or with anti-CR3 (**solid line**) MAbs. These experiments are representative of three different experiments. **B**. Binding of E-C3bi on THP-1 cells preincubated either with RPMI, 10 μM HB-19, 10 μM 9R or 20 μg anti-CR3 was determined by counting 100–200 cells by phase-contrast microscopy. Data show mean from three independent experiments ± SD values indicated as error bars.

We tested whether HB-19 could affect CR3 expression by flow cytometry and E-C3bi rosette formation (Fig 3). FACS analysis with anti-CR3 MAb gave a mean intensity for THP-1 cells preincubated with 10 μM HB-19 (Fig 3A1) or 10 μM 9R (Fig 3A2) of 27.37 and 24.54, respectively (as compared to 6.43 with anti-GAPDH). Rosette formation between E-C3bi and THP-1 cells (Fig 3B) was enumerated by phase-contrast microscopy. When THP-1 cells were preincubated with 10 μM HB-19 or 10 μM 9R, 48% and 51% ± 5% cells, respectively, were found with attached E-C3bi, as compared to 50% ± 6% cells with RPMI. When cells were preincubated with 20 μg anti-CR3, only 25% ± 3% cells formed rosettes with E-C3bi. These results suggested that HB-19 does not affect CR3 activity.

Preliminary experiments were also performed either with adherent THP-1 cells, obtained after treatment for 48 h with 200 ng/ml phorbol myristate acetate or with monocyte-derived macrophages (MDM), isolated from peripheral blood mononuclear cells as described [5] A similar inhibitory effect at 10 μM HB-19 of 70% and 65% was recorded for adherent THP-1 cells and MDM, respectively (data not shown).

Altogether, these results showed that nucleolin is involved in LVS interaction with human monocyte-like THP-1 cells and that LVS binding to nucleolin appears to be mediated through its C-terminal domain, as HB-19 binds RGG domain. The additive inhibitory effect of HB-19 on anti-CR3 further suggested that nucleolin interaction with LVS might be complementary to CR3 – C3 interaction.

### Identification of the bacterial ligands for nucleolin

Translation factor EF-Tu has been identified by analysis of the anti-*F. tularensis *immunoproteome as one of the major LVS antigenic protein in murine infection [32], and as an immunoreactive antigen present in membrane proteins enriched fraction of LVS in human infection [33].

**Figure 4:**
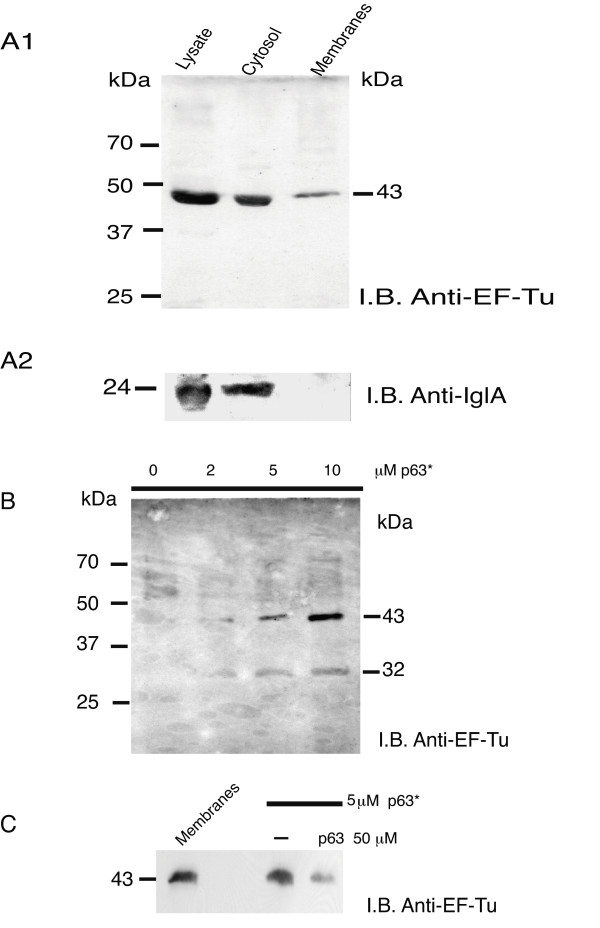
**Identification of bacterial ligands for nucleolin**. **Part A**. Samples were prepared as outlined in Experimental Procedures, normalized to 50 μg proteins per lane before separation by SDS-PAGE. Immunoblotting (I.B.) was performed either with anti-EF-Tu Ab diluted 1/100,000 (**part A1**) or anti-IglA Ab diluted 1/5,000 (**part A2**). **Part B**. 500 μg LVS membrane proteins were incubated with different concentrations of biotinylated p63 peptide (p63*). Complexes, formed between bacteria proteins and biotinylated p63 peptide, were isolated by purification using avidin-agarose. Purified proteins were analyzed by SDS-PAGE. Immunoblotting (I.B.) was performed with anti-EF-Tu Ab diluted 1/100,000. Mobility of marker proteins is indicated on left side. **Part C**. Specific binding of EF-Tu present in LVS membranes with RGG domain of nucleolin. 500 μg LVS membrane proteins were preincubated without (**lane **-) or with 50 μM unlabeled p63 peptide, before incubation with 5 μM biotinylated p63 peptide (p63*). Complex formed between EF-Tu present in bacteria membrane proteins and biotinylated p63 peptide was analyzed by immunoblotting with anti-EF-Tu Ab diluted 1/100,000. In control, 50 μg LVS membrane proteins were run directly.

We made the assumption that LVS EF-Tu, possibly present on LVS cell surface, could be a ligand for nucleolin and bind to its C-terminal domain. To test this hypothesis, we first prepared a specific anti-EF-Tu antibody. Sequence of LVS gene encoding EF-Tu (FTL_1751) was obtained by *in silico *analysis of LVS genome [34]. LVS EF-Tu presents 99.7% amino acid identity with EF-Tu from type A strain, *F. tularensis *Schu S4 (FTT_0137). Purified recombinant EF-Tu, tagged either with polyhistidine tag (His-EF-Tu) or with glutathione S-transferase protein (GST-EF-Tu), were prepared in *E. coli*. Both recombinant proteins had expected apparent molecular mass (MW) on SDS-PAGE i.e. 43 kDa for His-EF-Tu and 70 kDa for GST-EF-Tu (data not shown). We prepared polyclonal anti-EF-Tu antibody by immunizing mice with His-EF-Tu recombinant protein. The specificity of anti-EF-Tu Ab was demonstrated, as only one protein, characterized by an apparent Mr of 43 kDa, was recognized by anti-EF-Tu Ab, among all proteins present in a whole bacteria cell lysate (Fig 4A1). After cell fractionation of LVS, EF-Tu was found expectedly in the bacterial cytoplasmic fraction [17], but also in the membrane fraction. In contrast, the cytosolic protein IglA was exclusively localized in the cytoplasmic fraction [20] (Fig 4A2).

We then assessed whether EF-Tu present in LVS membrane fractions bound to p63 RGG carboxy-terminal domain of nucleolin, by a pull-down assay. Solubilized LVS membrane proteins were incubated with increasing amounts of biotinylated p63 peptide. The bacterial protein(s)-biotinylated p63 peptide complexes, recovered after passage through avidin-sepharose columns were probed with anti-EF-Tu antibody (Fig 4B). The 43 kDa EF-Tu was detected in LVS membranes by anti-EF-Tu Ab. Increasing amounts of EF-Tu were recovered with increasing concentrations of biotinylated p63, with maximum recovery obtained at 10 μM peptide concentration. An additional product of 32 kDa compatible with a proteolytic degradation product of the protein was also recovered from bound proteins. Similar truncated fragment has been described for EF-Tu of other species [35]. Specificity of EF-Tu binding to 5 μM biotinylated p63 was confirmed by inhibition of this interaction by 10-molar excess of unlabeled p63 (Fig 4C).

### Interaction of recombinant EF-Tu with C-terminal RGG domain of nucleolin is specific

**Figure 5:**
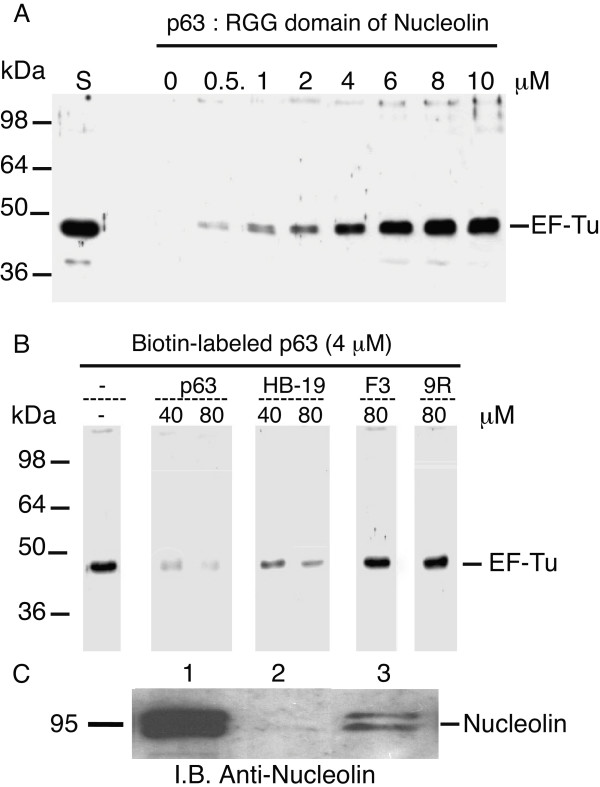
**Recombinant EF-Tu specifically interacts with RGG carboxy-terminal domain of nucleolin**. **A**. P63 peptide, corresponding to RGG domain of nucleolin binds in dose-dependent manner to recombinant EF-Tu. Aliquots containing 1 μg recombinant His-EF-Tu were incubated with different concentrations of biotin-labeled p63 peptide. Complex formed between His-EF-Tu and biotin-labeled p63 peptide was isolated by purification using avidin-agarose. Purified proteins were analyzed by SDS-PAGE. **Lane S **shows purified preparation of 0.2 μg His-EF-Tu. Presence of EF-Tu was detected by immunoblotting using anti-EF-Tu Ab diluted 1/100,000. **B**. Specific binding of recombinant EF-Tu with RGG domain of nucleolin. Aliquots containing 1 μg of His-EF-Tu were preincubated without (**lane **-) or with 40 or 80 μM unlabeled p63 peptide before further incubation with 4 μM biotin-labeled p63 peptide. Biotin-labeled p63 peptide (4 μM) was also preincubated with HB-19 (40 or 80 μM), 80 μM F3 or 80 μM 9Arg (9R) peptides before further incubation with 1 μg His-EF-Tu. Complexes formed between His-EF-Tu and biotin-labeled p63 peptide were isolated and analyzed by immunoblotting as in section **A**. On left is position of molecular weight protein markers. On right is position of 43 kDa His-EF-Tu. **C**: 5 μg GST (**lane 2**) or GST-EF-Tu (**lane 3**) bound on glutathione-Sepharose beads were incubated with THP-1 solubilized membrane proteins (500 μg). After extensive washes, bound proteins were run on SDS-PAGE with, in control, 50 μg proteins solubilized from THP-1 membranes, run directly without incubation with beads (**lane 1**). Immunoblotting was performed with anti-nucleolin MAb, diluted 1/10,000. Estimated molecular mass (in kDa) of nucleolin is indicated on left of black line.

Specificity of EF-Tu interaction with nucleolin was further confirmed by two reciprocal pull-down assays (Fig 5). The first pull-down assay evaluated capacity of recombinant His-EF-Tu to bind to p63 peptide. Recombinant EF-Tu bound in dose-dependent manner to biotin-labeled p63 peptide (Fig 5A) with maximum binding occurring at 8 μM. Specificity of this binding was demonstrated by its inhibition with a 10 to 20-molar excess of unlabeled p63 peptide (Fig 5B). Effect of HB-19 pseudopeptide and F3 or 9R peptides was also tested on EF-Tu interaction with RGG domain. A 10 to 20-molar excess HB-19 markedly inhibited binding of biotin-labeled p63 peptide to EF-Tu, while a 20-molar excess of F3 or 9R peptides that do not interact with nucleolin had no effect (Fig 5B).

The second pull-down assay showed that nucleolin, present in solubilized proteins from THP-1 cell membranes, interacted with recombinant EF-Tu (Fig 5C). Human solubilized membrane proteins were incubated with glutathione-sepharose bound GST-EF-Tu or GST as control. A protein doublet at 95 kDa, corresponding to human nucleolin, was detected by anti-nucleolin MAb only in GST-EF-Tu sample (**lane 3**). Nucleolin was not detected with GST (**lane 2**). **Lane 1 **shows the amount of nucleolin detected in 50 μg total membrane proteins. Blotting with polyclonal anti-GST demonstrated that same amounts of GST or GST-EF-Tu were loaded in the gel (data not shown). These results demonstrated that "in vitro" a) recombinant EF-Tu interacts with nucleolin present in THP-1 cell membranes; b) RGG p63 carboxy-terminal domain of nucleolin interacts with EF-Tu either recombinant or present in membranes of LVS.

### EF-Tu is localized on bacterial surface

**Figure 6:**
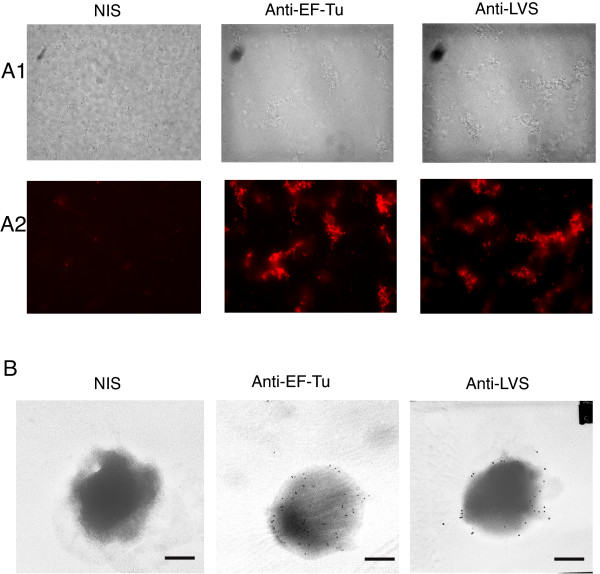
**Elongation factor Tu is localized on external side of *F. tularensis *LVS membranes**. **Part A**. Fluorescence microscopy analysis of LVS pellet incubated either with NIS (from rabbit or from mice), anti-EF-Tu or anti-LVS Abs all diluted 1/10,000. Bright fields (**part A1**) or fluorescence microscopy (**part A2**) of 15 fields for each group were observed at 63× magnification. Images are representative of 3 different experiments. **Part B**. Electron micrographs showing attachment of gold spheres to surface of LVS incubated either with anti-EF-Tu or rabbit anti-LVS Abs and examined by nc-IEM. Control micrograph of LVS incubated with murine NIS shows no beads on bacteria cell surface. Electron microscopy was observed at 20,000× magnification. Size bars equal 500 nm.

We then tested the surface exposition of EF-Tu, which was detected in LVS membrane fractions, by fluorescence and electron microscopy (Fig 6). Immunofluorescence analysis (Fig 6A) of intact LVS bacteria gave positive labeling with anti-EF-Tu Ab, as with anti-LVS Ab. No signal was obtained with rabbit anti-nucleolin (see Additional file 1) and either NIS (rabbit or mouse) or anti-nucleolin Mab (data not shown). Immunoelectron microscopy of LVS (Fig 6B) showed positive immunogold staining of surface of intact LVS both with anti-EF-Tu Ab and with control anti-LVS Ab. No staining was found with control NIS. These data strongly suggested that a fraction of EF-Tu is exposed at surface of LVS and may therefore interact with cell-surface associated nucleolin.

### Co-localization of cell-surface expressed nucleolin with EF-Tu of F. tularensis

**Figure 7:**
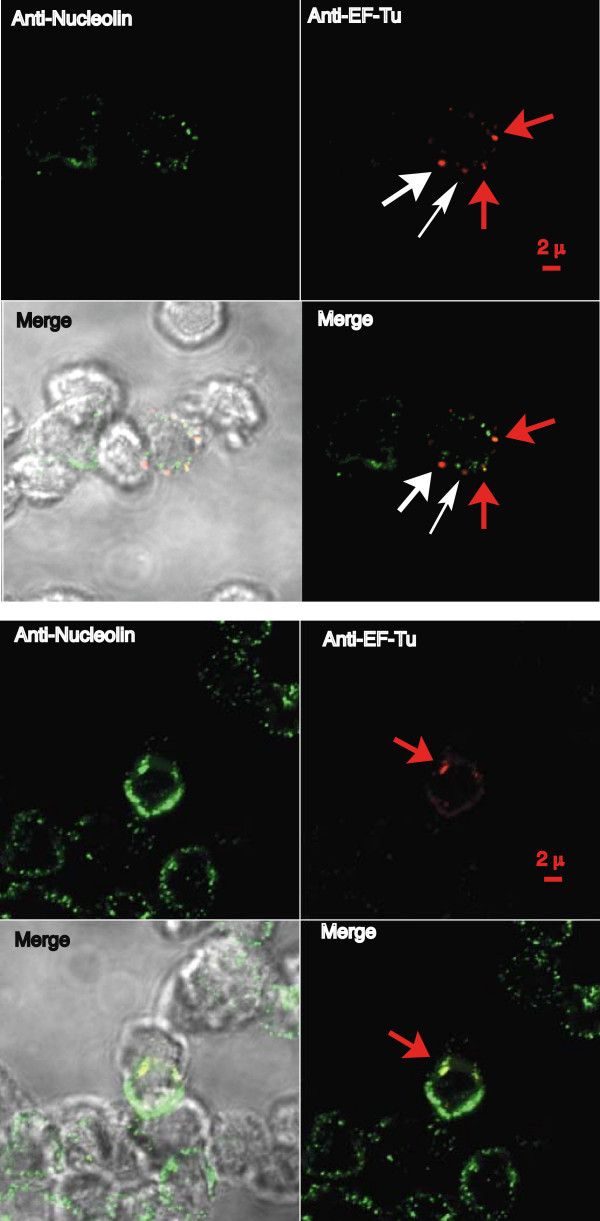
**Cell surface nucleolin co-localizes with LVS elongation factor Tu**. THP-1 cells were incubated for 30 min with opsonized LVS and their interaction was observed by confocal microscopy at 63× magnification. Human cell surface was labeled with rabbit anti-nucleolin Ab diluted 1/200. Bacteria were labeled with murine anti-EF-Tu Ab diluted 1/2,000. Merging was observed with 3 × Zoom either with fluorescence light (**right panel**) or as bright field (**left panel**). **Red arrows **indicate colocalization of LVS with nucleolin present on cell surface. **White arrows **indicate LVS bound on cell surface in absence of nucleolin. These two photos are representative of five different experiments.

Localization of EF-Tu at LVS surface and of nucleolin on human cell surface prompted us to investigate by confocal microscopy, whether they could co-localize on THP-1 cell surface (Fig 7). For that purpose, THP-1 cells were first incubated with LVS for 30 min at 37°C, before further incubation with polyclonal anti-nucleolin Ab. Under these conditions, anti-nucleolin Ab labels cell surface and not intracellular nucleolin (see Fig 1A). This polyclonal anti-nucleolin Ab was shown not to cross-react with LVS (see Additional file 1). Binding of opsonized bacteria was monitored with murine anti-EF-Tu Ab, which specifically recognized the 43 kDa EF-Tu protein (see Fig 3). Labeling of infected cells with anti-nucleolin Ab gave a lower signal, than when cells are not infected, as already shown by FACS analysis (see Fig 1). Merging of nucleolin with LVS EF-Tu was observed only when EF-Tu present on LVS bacteria met clustered patches of nucleolin (**see red arrows**). No merging of LVS was observed when nucleolin was not present (**see white arrows**). To quantify the number of LVS particles co-localizing with nucleolin, we used LVS-GFP particles (see Methods). Merging of LVS-GFP with nucleolin was observed in these conditions (see Additional file 2) and quantified with Imaris program. Regions of interest (ROI) indicated a percentage of 21% ± 5% LVS-GFP particles co-localizing with nucleolin on more than 500 observed cells.

### Anti-EF-Tu antibody inhibits binding to and infection of human monocyte-like THP-1 cells by LVS

**Figure 8:**
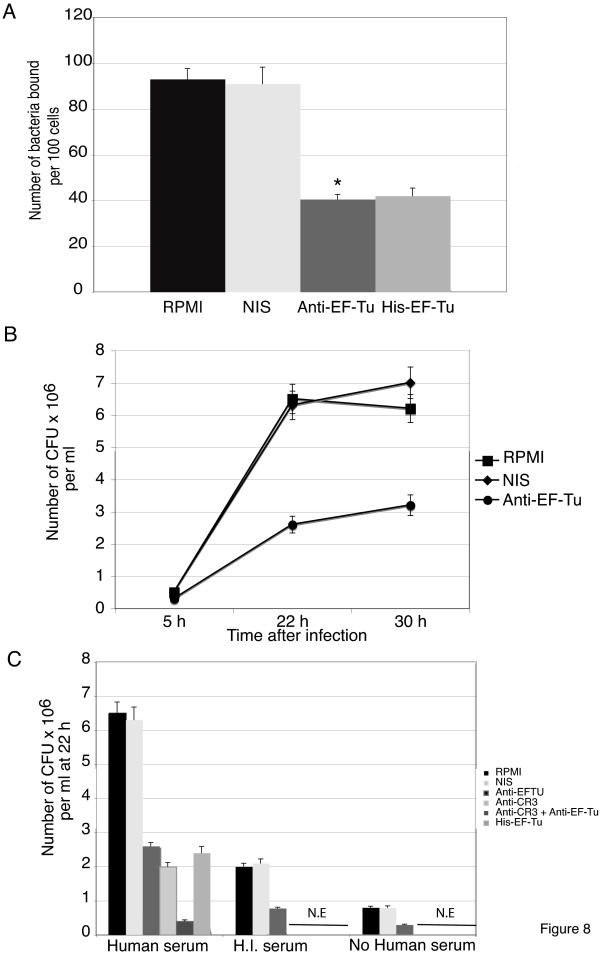
**Elongation factor Tu of LVS participates in binding to human monocyte-like THP-1 cells and in their infection**. **A**: THP-1 cells were infected for 30 min by opsonized LVS-GFP that had been pre-incubated either with RPMI, NIS or anti-EF-Tu Ab (diluted 1/2,000). THP-1 cells were also pre-incubated with 50 μg His-EF-Tu and then infected for 30 min by opsonized LVS-GFP pre-incubated with NIS. Fluorescence microscopy was analyzed at 63× magnification. Fifteen fields containing average of 100 cells were examined to quantify number of bound bacteria. Results shown are means ± SD from three independent experiments. *, p < 0.01 for group of bacteria treated with anti-EF-Tu Ab compared to group incubated with RPMI. **B: **Intracellular replication of LVS. THP-1 cells were infected for 30 min with opsonized LVS that were first incubated either with RPMI (■), mouse NIS (◆) or anti-EF-Tu Ab (●), both diluted 1/2,000. Cells were then washed with RPMI containing gentamicin and further incubated in RPMI-FCS and gentamicin for indicated times. Quantification of intracellular bacteria was performed as described in Methods. Results shown are means from five independent experiments, each in triplicate ± SD indicated by error bars. **C: **LVS were incubated either with **Human serum**, **H.I. serum **or with **No Human serum**. LVS in these different conditions were further incubated either with RPMI, mouse NIS or anti-EF-Tu Ab, both diluted 1/2,000. THP-1 cells were then infected by the different LVS preparations. THP-1 cells, pre-incubated with anti-CR3 MAb were infected with opsonized LVS that had been pretreated or not by anti-EF-Tu Ab. THP-1 cells were also preincubated with 50 μg His-EF-Tu before infection by opsonized LVS. Cells were washed in RPMI with gentamicin and incubated in RPMI-FCS and gentamicin for 22 h. Quantification of intracellular bacteria was performed as described in Methods. **N.E. (not examined)**: anti-CR3 MAb, anti-CR3 MAb and anti-EF-Tu Ab and His-EF-Tu samples were not tested in **H.I serum **and **No human serum **conditions. Results shown are means from five independent experiments, each in triplicate ± SD indicated by error bars.

Involvement of EF-Tu in binding of LVS on THP-1 cells was assessed by fluorescence analysis, using LVS-GFP bacteria and THP-1 cells (Fig 8A). THP-1 cells were therefore incubated with opsonized LVS-GFP bacteria that had been pre-incubated either with RPMI, NIS or anti-EF-Tu Ab. A maximum binding of 95 LVS-GFP bacteria per 100 human cells was recorded, in presence of RPMI or NIS. When surface-exposed EF-Tu was blocked by anti-EF-Tu Ab, number of bacteria bound per 100 cells was decreased by 58%. Consistent with this, soluble recombinant EF-Tu also decreased the number of bacteria bound on cells. Indeed, incubation of THP-1 cells with 50 μg His-EF-Tu before infection with opsonized LVS-GFP decreased LVS binding by 57%. We then analyzed effect of anti-EF-Tu Ab on intracellular replication of LVS after THP-1 cell infection by opsonized LVS (Fig 8B). At 4 h, the 5 ± 0.6 × 10^5 ^CFU/ml obtained in presence of RPMI were decreased to 1 ± 0.2 × 10^5 ^CFU/ml with anti-EF-Tu Ab. Maximum of intracellular bacterial multiplication (6.5 × 10^6 ^CFU/ml) was reached between 22 to 30 h after infection. A maximum decrease (60%) in bacterial number was observed at 22 h when bacteria were pre-incubated with anti-EF-Tu Ab. This corresponded to a decrease from 11 to 4 intracellular bacteria per cell. All these experiments were performed in presence of normal human serum. We therefore analyzed at 22 h whether anti-EF-Tu Ab had a similar inhibitory effect on level of infection when LVS was incubated in presence of heat-inactivated (H.I) serum or in absence of serum (Fig 8C). A lower infection of THP-1 cells by LVS was observed, in presence of H.I. serum and in absence of serum (3 and 1.5 intracellular bacteria, respectively), consistent with earlier observations [6,31]. However, even in these conditions, LVS preincubation with anti-EF-Tu Ab resulted in 60% inhibition of infection. Moreover, when human cells were pre-incubated with anti-CR3 MAb and infected by opsonized bacteria that had been pretreated with anti-EF-Tu Ab, maximal decrease of 94% in infection was observed (corresponding to less than 1 bacterium per cell).

All these results indicated that, in addition to C3, EF-Tu was important for LVS infection of THP-1 cells through nucleolin. Specific involvement of EF-Tu in LVS infection was also supported, by the 67% decrease in infection observed using 50 μg recombinant EF-Tu. Altogether, our results show that: i) EF-Tu is involved in LVS binding to – and infection of – human THP-1 monocyte-like cells; ii) in spite of requirement for CR3-C3 interaction for LVS infection, nucleolin – EF-Tu interaction is distinct and should be important to consider.

## Discussion

The receptor-ligand pathways involved in entry of *F. tularensis *into human monocytes are still not clearly understood. Here, for the first time, we have characterized protein partners of a bacteria-host cell interaction, which permits entry of LVS into human monocyte-like THP-1 cells and their subsequent infection. We showed that elongation factor EF-Tu expressed at bacterial surface interacts with nucleolin, expressed on surface of human monocyte-like THP-1 cells. Blockade of this interaction, either with nucleolin-binding pseudopeptide HB-19 or with anti-EF-Tu Ab, results in inhibition of LVS bacteria binding to human THP-1 cells and subsequent inhibition of bacterial infection.

Presence of nucleolin at surface of THP-1 monocyte-like cells was demonstrated by fluorescence microscopy and FACS analysis. The fact that nucleolin expression was decreased following LVS infection suggests that nucleolin plays a role in LVS adhesion and does affect entry process and infection. Nucleolin has three main structural domains [9]: 1) N-terminal domain, containing several long stretches of acidic residues; 2) central globular domain, containing four RNA binding domains (RBDs); and 3) C-terminal domain containing nine repeats of tripeptide motif arginine-glycine-glycine (RGG domain = 63 amino acid residues). We demonstrated the important role of nucleolin in LVS infection by using nucleolin-specific peptides. HB-19 pseudopeptide, which specifically binds the RGG C-terminal domain of nucleolin, inhibited both binding and infection of human monocyte-like THP-1 cells infection by LVS. The basic 9R peptide, which does not bind to nucleolin [11,23] or the F3 tumor-homing peptide, which binds on N-terminal domain of nucleolin [25], had no effect on LVS infection. These results confirmed the specific role of nucleolin in LVS infection and demonstrated that nucleolin interacts with LVS through its carboxy-terminal RGG domain.

We used THP-1 cells in suspension for our experiments. The limited amount of *ca*. 90 bacteria bound per 100 THP-1 monocyte-like cells we observed was very similar to that previously found with macrophages and MDM (67–75 Ft/100 cells) [8]. Nevertheless, phagocytic activity of non-adherent THP-1 cells showed to be sufficient to internalize bacteria and promote active intracellular multiplication, since an average of ten bacteria per infected cell was found at 22 h after infection. In preliminary experiments performed either with adherent THP-1 cells, or with MDM, a similar inhibitory effect of HB-19 was recorded with these cells. In view of this result, further investigations are required to evaluate the implication of nucleolin in LVS infection of phagocytic cells other than THP-1 cells.

RGG domain of nucleolin constitutes the binding site for various ligands such as subset of ribosomal proteins [12,36,37], midkine, pleiotrophin, lactoferrin, and even HIV particles [23,37-40]. P63 synthetic peptide, which corresponds to RGG domain of nucleolin, allowed us to characterize the bacterial partner of nucleolin, elongation factor Tu. The C-terminal RGG domain bound both intact bacteria and recombinant EF-Tu. Direct interaction of nucleolin with EF-Tu was also demonstrated by co-localization on human cell surface of both proteins and by a pull-down assay where solubilized nucleolin interacted with recombinant GST-EF-Tu.

Human nucleolin therefore serves as surface receptor for EF-Tu presented by LVS and this interaction plays a major role in binding of LVS and its subsequent infection of human monocyte-like THP-1 cells. Nucleolin-specific HB-19 pseudopeptide, anti-EF-Tu Ab and recombinant EF-Tu inhibited both binding and infection of human monocyte-like THP-1 cellss. Coating bacteria with anti-EF-Tu Ab did not confer subsequent phagocytosis by Fc receptors as a lower number of CFU was obtained. We always used the same batch of fresh human serum in all experiments to avoid differential effect due to donor-dependent FcγR involvement on opsono-phagocytosis. Indeed, presence of a low concentration of antibodies to *Francisella *in non-immune human serum, which will create ligands for Fc receptors, has been previously described [6].

Fate of surface nucleolin after its interaction with LVS, observed by FACS analysis and its subsequent internalization, remain to be investigated, inasmuch as in all microscopy and flow cytometry experiments, primary Abs were added to living cells at room temperature where pinocytosis still occurs. It has been already suggested that internalization of nucleolin-binding ligands occur via lipid rafts [23,24,37,39]. While surface nucleolin is readily solubilized by treatment of cells with non-ionic detergent, it becomes detergent-resistant after ligand binding to cells and is recovered along detergent insoluble membrane microdomains containing lipid raft. Internalization of LVS through nucleolin was blocked by cytochalasin D (data not shown). This result is consistent with previous data, which showed that infection by LVS of human monocyte-like THP-1 cells was completely blocked by cytochalasin D [5,13].

A fraction of EF-Tu was localized at the LVS surface. This is the first protein of *F. tularensis *to be characterized as a specific ligand involved in bacterial entry into human monocyte-like THP-1 cells. Interestingly, recent studies have unraveled an unexpected key role for EF-Tu in microbial pathogenesis, e.g. i) EF-Tu, located at cell surface of *Lactobacillus johsonii*, mediates its attachment to human intestinal cells and mucins [41]; ii) EF-Tu is one of the six most abundant mycobacterial proteins, whose expression increases after phagocytosis by macrophages [42]; iii) EF-Tu of *Listeria monocytogenes *is target for a serine-threonine phosphatase, which is involved in virulence of this pathogen [35]; iv) EF-Tu is present in purified *Anaplasma marginale *outer membranes that induce complete protection against infection [43]. A very recent report [44] also demonstrates that bacterial surface Tuf of *P. aeruginosa *acts as virulence factor and binds human complement factor H and plasminogen.

EF-Tu belongs to the growing list of cytoplasmic proteins identified on bacterial surface. At present, the mechanisms of their surface localization and attachment to the bacterial envelope remain unclear. Indeed, these proteins (including EF-Tu, pyruvate kinase, glyceraldehyde-3-phosphate dehydrogenase and ribosomal proteins) do not contain signal peptides that could direct them into classical secretory pathways and do not possess any obvious envelope-associating motifs. Localization of EF-Tu at the bacterial surface may be explained by its three-dimensional structure. Indeed, EF-Tu protein consists of three domains: an alpha/beta domain (residues 1 to 200), which contains the binding site of the GDP cofactor and two all-beta domains (residues 209 to 299 and 300 to 393), which belong to the tertiary structural class of antiparallel beta-barrels [45]. At this stage, it is tempting to imagine a model somewhat similar to that described in gram-negative bacteria for the autotransporter pathway [46], where the C-terminal part of the precursor forms a porin-like structure within the outer membrane. The N-terminally attached passenger domain is ultimately translocated to the cell surface through this pore. However, in the autotransporter pathway, an N-terminal signal peptide drives the transport of the precursor protein across the inner membrane. Various "environmental" signals might trigger translocation of a subpopulation of cytoplasmic proteins to bacterial membrane surface. Alternatively, conformational changes might allow membrane localization of otherwise cytoplasmic proteins, and provide them alternative or "moonlighting" functions in this compartment.

Therefore, our results support a "moonlighting function" for bacterial EF-Tu, already described for its eukaryotic analog, the EF1 elongation factor. Indeed, eukaryotic elongation factors have been shown to be concerned in various important cellular processes or serious diseases, including translational control, signal transduction, cytoskeletal organization, apoptosis, adult atopic dermatitis, oncogenic transformation, nutrition, and nuclear processes such as RNA synthesis and mitosis [47,48].

EF-Tu also interacted with nucleolin in presence of inactivated C3 and even in absence of C3. While HB-19 presented an additive inhibitory effect on anti-CR3, it did not interfere with CR3 expression and/or its activity. This suggested a receptor pathway independent of CR3. Therefore, by mediating infection by *F. tularensis*, EF-Tu – nucleolin interaction may play a role in immuno-compromised patients where C3 deficiencies are associated with higher susceptibility to severe infections [49].

The quantitative importance of nucleolin as a receptor pathway compared to the CR3/C3 pathway has to be assessed by further experiments. Twenty-one percent ± 5% opsonized LVS co-localized with nucleolin, as quantified by confocal analysis while 78% bacteria had their binding inhibited by HB-19. This difference may be explained by the difference in sensitivity of the diverse methods and conditions used. Indeed, a similar difference was observed for CR3/C3 interaction. While infection by LVS of THP-1 cells preincubated with anti-CR3 was decreased by 70%, 50% of human THP-1 cells formed rosettes with E-C3bi, with only 50% of these rosettes inhibited by anti-CR3 preincubation of the cells.

## Conclusion

EF-Tu has been reported to act as stimulator of proinflammatory response in presence of soluble CD14 in Arabidopsis plants [50,51]. EF-Tu was described as acting as pathogen-associated molecular pattern (PAMP) for innate immune system of both animals and plants. After an initial high affinity binding to its receptor, EF-Tu from Arabidopsis induced elements of signal transmission, including activation of MAPK. By similarity to these recent observations, it is tempting to suggest that interaction of EF-Tu with human nucleolin might be involved in innate immune response to *F. tularensis*, by triggering transduction signals. Indeed, previous reports on macrophage responses to LVS infection included production of inflammatory cytokines and activation of NF-κB activation [52,53]. Since phagosomal escape and intra-cytosolic multiplication of LVS in infected monocytes are very similar to those of human pathogenic *F. tularensis *ssp *tularensis *[3,4], the mechanism of entry into monocyte-like cells, involving interaction between EF-Tu and nucleolin, might be similar in the two subspecies. Thus, use of either nucleolin-specific pseudopeptide HB-19 or recombinant EF-Tu, could provide attractive therapeutic approaches for modulating *F. tularensis *infection.

## Abbreviations

CR3: complement receptor type 3; EF-Tu: elongation factor Tu; LVS: live vaccine strain; MOI: Multiplicity of Infection; NIS: non immune serum; HB-19: 5 [Lys (CH_2_N)Pro-Arg]-template-assembled synthetic peptide; 9R: the 9Arg peptide, nine-D-arginine peptide; F3 peptide: the tumor-homing peptide of 34 amino acid residues; the p63 peptide: the synthetic peptide corresponding to the last 63 amino acid residues of human nucleolin.

## Authors' contributions

MB designed, carried out experiments and drafted the manuscript. AGH designed, carried out experiments and critically revised the manuscript. KM critically revised the manuscript for important intellectual content. JPB synthesized the different peptides. MD performed the electron microscopy experiments. AC designed experiments and revised critically the manuscript for important intellectual content.

## Supplementary Material

Additional file 1 Rabbit anti-nucleolin Ab does not recognize LVS. Fluorescence microscopy analysis of LVS pellet incubated either with rabbit anti-LVS or rabbit anti-nucleolin Abs and observed as bright fields (**part A1**) or fluorescence microscopy (**part A2**). Click here for file

Additional file 2 Cell surface nucleolin co-localizes with LVS. Interaction of THP-1 cells with opsonized LVS-GFP (**Left panel**, **green**) was observed by confocal microscopy on single optical sections (Z = 0.99 μm). Human cell surface was labeled with anti-nucleolin MAb and Alexa-Fluor 568 GAM (**red**) and cell nuclei were labeled with DAPI (**blue**) (**Center panel)**. **Right panel **is a merged image (**yellow**). **Red arrows **indicate co-localization of LVS with nucleolin present on cell surface. Scale bar = 30 μm. Click here for file
