# Supplementary figures and images for: A novel receptor – ligand pathway for entry of Francisella tularensis in monocyte-like THP-1 cells: interaction between surface nucleolin and bacterial elongation factor Tu
Source: BMC Microbiol. 2008 Sep 12;8:145. doi: 10.1186/1471-2180-8-145 (PMC2551611; doi:10.1186/1471-2180-8-145)

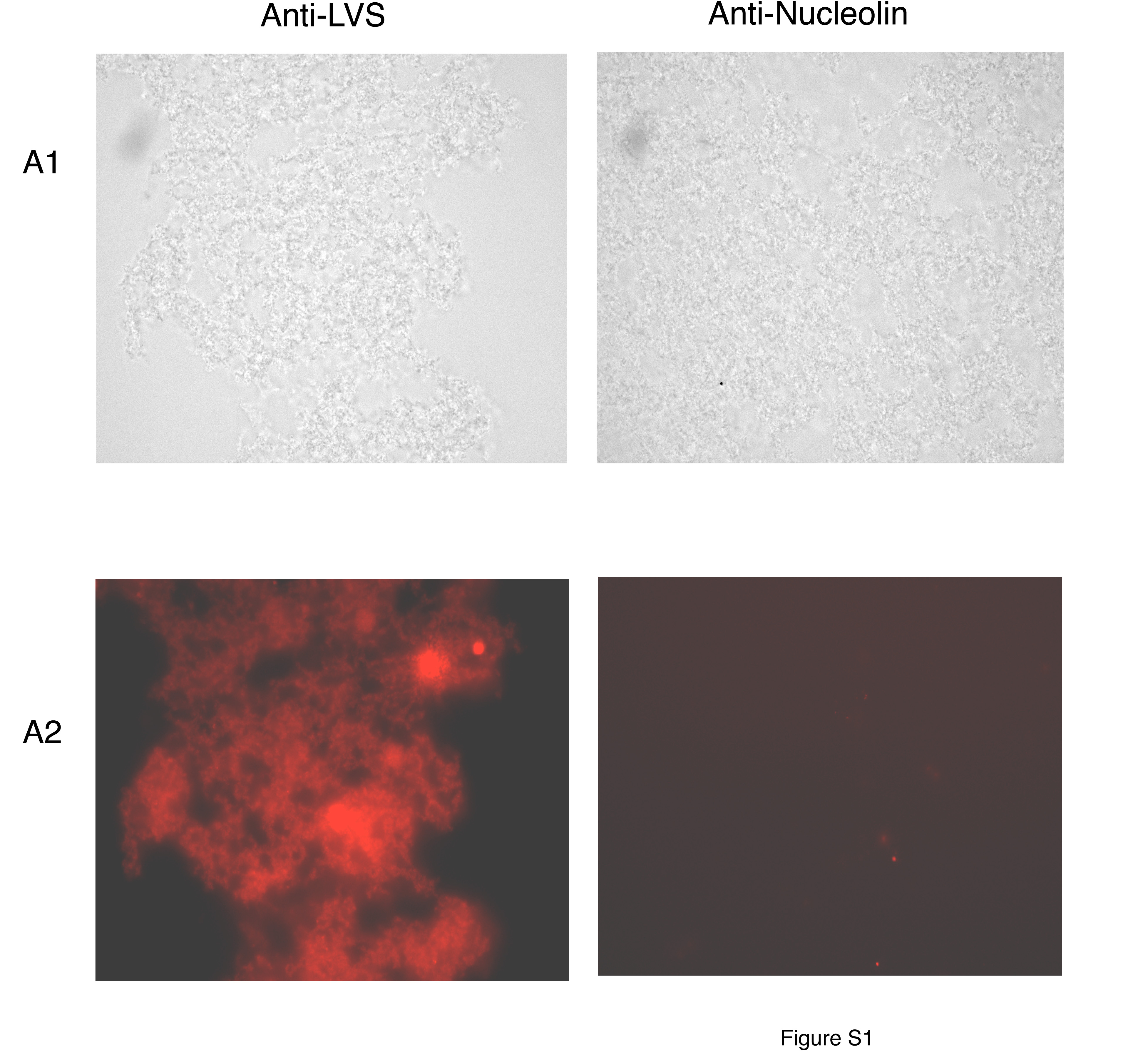

Supplement: Additional file 1 — Rabbit anti-nucleolin Ab does not recognize LVS. Fluorescence microscopy analysis of LVS pellet incubated either with rabbit anti-LVS or rabbit anti-nucleolin Abs and observed as bright fields (part A1) or fluorescence microscopy (part A2). [file 1471-2180-8-145-S1.jpeg]

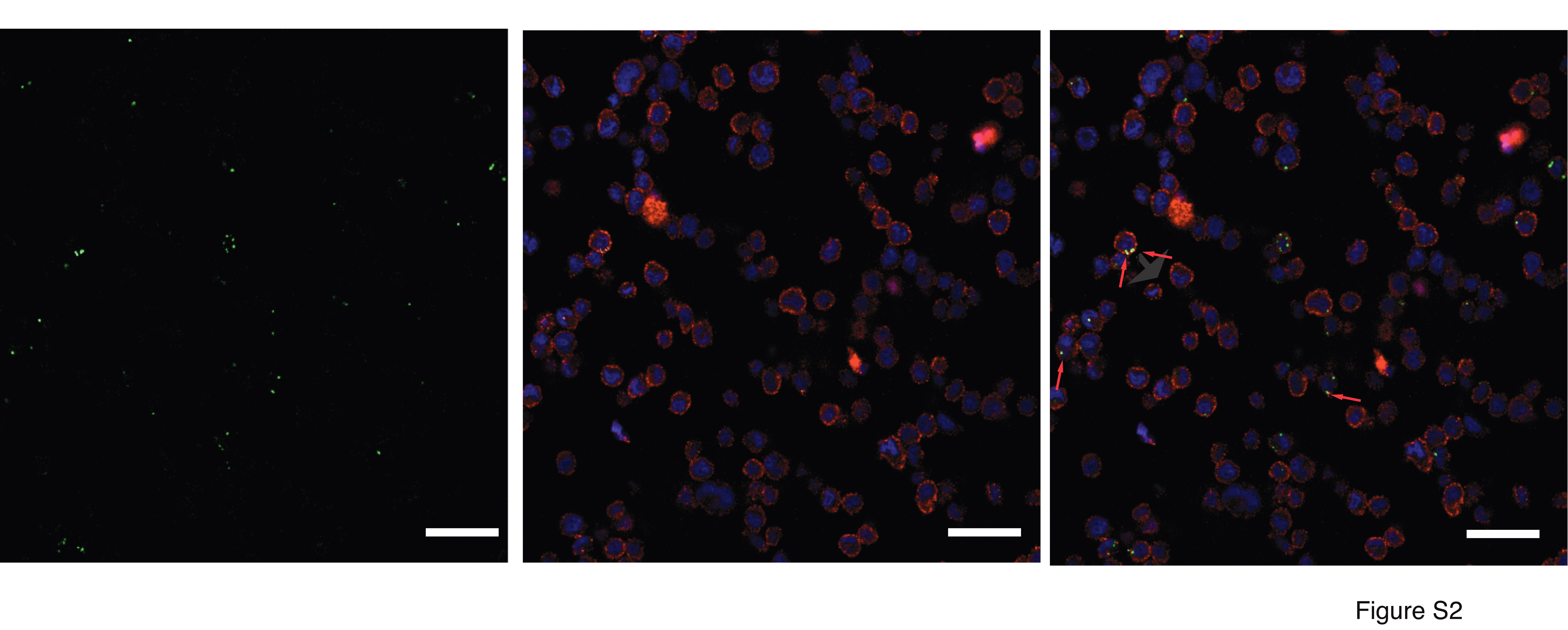

Supplement: Additional file 2 — Cell surface nucleolin co-localizes with LVS. Interaction of THP-1 cells with opsonized LVS-GFP (Left panel, green) was observed by confocal microscopy on single optical sections (Z = 0.99 μm). Human cell surface was labeled with anti-nucleolin MAb and Alexa-Fluor 568 GAM (red) and cell nuclei were labeled with DAPI (blue) (Center panel). Right panel is a merged image (yellow). Red arrows indicate co-localization of LVS with nucleolin present on cell surface. Scale bar = 30 μm. [file 1471-2180-8-145-S2.jpeg]
